# Considering patient perspectives in economic evaluations of health interventions

**DOI:** 10.3389/fpubh.2023.1212583

**Published:** 2023-10-09

**Authors:** Rui Fu, Vivian Ng, Michael Liu, David Wells, Emre Yurga, Eric Nauenberg

**Affiliations:** ^1^Department of Otolaryngology—Head & Neck Surgery, Sunnybrook Research Institute, University of Toronto, Toronto, ON, Canada; ^2^Institute of Health Policy, Management and Evaluation, University of Toronto, Toronto, ON, Canada; ^3^Roche Diagnostics, Laval, QC, Canada; ^4^Harvard Medical School, Harvard University, Boston, MA, United States; ^5^The Canadian Agency for Drugs and Technologies in Health (CADTH), Nanaimo, BC, Canada; ^6^Ontario Ministry of Health, Toronto, ON, Canada

**Keywords:** health economics, cost-effectiveness analysis, economic evaluations, patient-centered care, patient preference

## Abstract

Current guidelines for evaluating the cost-effectiveness of health interventions commonly recommend the use of a payer and/or a societal perspective. This raises the concern that the resulting reimbursement decision may overlook the full spectrum of impacts and equity considerations. In this paper, we argue that a potential solution is to supplement a societal- or payer-perspective economic evaluation with an additional evaluation accounting for exclusively the patient perspective. We present five categories of health interventions for which a patient-perspective analysis may be informative including those (1) that cross the definitional boundary between drugs and non-drug technologies; (2) affect patient adherence to protocol; (3) represent revolutionary treatments for genetic disorders; (4) with an incremental cost-effectiveness ratio involving slightly less effective, but substantially less costly, than the current standard; and (5) have been previously approved for funding but now being targeted for potential delisting or disinvestment. Real-world examples are discussed in detail. Lived experience individuals were invited to provide vignettes. Discussions are provided regarding how to incorporate patient inputs to improve patient-centered decision-making.

## 1. Introduction

Economic evaluations, as reviewed by Turner et al. in an earlier issue of *Frontiers in Public Health*, refer to a type of analysis that simultaneously assesses the costs and effects of alternative interventions to ensure value for resources expended from various perspectives (1). Although there have been attempts to address equity considerations in cost-effectiveness analyses (CEAs) through the development of distributional and extended cost-effectiveness methods, they stated that “this is still an area that needs attention regarding practical implementation with regards to informing resource allocation decisions in global health” (1). This shortfall emanates, in part, from inadequate analysis of the burdens placed on individuals and families from the introduction of new health interventions.

Commonly, only the societal and/or the payer perspective are recommended by health technology assessment (HTA) agencies. At the national level, the latter is endorsed by countries including Australia, the UK and Canada (2). Other countries (e.g., Thailand) have recommended a societal perspective while at least one—Norway—has limited it to exclude productivity gains and losses (2). The US has joined a small number of countries (e.g., Italy) recommending both perspectives as reference cases (3).

A pressing issue with such perspectives is that they may insufficiently highlight impacts on lived experience individuals, including patients and families/caregivers (henceforth “patients” for simplicity) (4). Although the societal perspective can capture some patient-borne financial impositions, they are aggregated with other costs, raising the concern that a favorable societal-perspective profile may not signal whether for patients an intervention is truly affordable, adherable or otherwise impactful (5). The affordability concern is accentuated when assessing innovative health interventions that straddle the definition of prescription drugs and non-drug technologies because the level of third-party coverage is *a-priori* unclear; therefore, patients may face substantial increases in out-of-pocket costs if an intervention is assigned to a category with lower levels of coverage than its predecessors (5). These interventions may also impose negative impacts on patient lives that would remain “hidden” within broader perspectives.

Herein, we argue that a potential solution—of particular interest to public health professionals—is to supplement a societal- or payer-perspective economic evaluation with an exclusive evaluation from the perspective of patients. While guidelines for incorporating patient inputs in HTA exist (6–8), a formal framework for conducting patient-perspective economic evaluations has never been formulated to our knowledge. As such, CEAs conducted with the patient perspective often vary in their coverage and definition of patient-borne costs (4). In this Perspective paper, we aim to provide a comprehensive discussion on the unique insights a patient-perspective economic evaluation could yield to aid reimbursement decision-making in healthcare.

## 2. Procedures

In this 2-part Perspective paper, we followed the Patient Preferences in Benefit–Risk Assessments during the Drug Life Cycle project and a subsequent focus group discussion with HTA representatives from Canada, Belgium and Germany (9, 10). In the first part of this paper, we formulated five categories of health interventions that could be used to flag when a supplementary analysis might be warranted through extensive discussion within our team. The issues covered include household-level affordability; adherence; unintended side effects; and burdens on daily living. A separate category was created for gene therapy as it has been regarded as a health technology particularly sensitive to the patient perspective (9, 10). Real-world examples are discussed.

To provide real-world perspectives on this topic, during December 2020, we invited lived experience individuals from the Patient and Family Advisors Network, a virtual network that comprises individuals covered under Ontario’s publicly funded healthcare insurance system who volunteer to serve as policy advisors for Ontario Health, to review the draft of this paper. Using a convenience sampling methodology, one researcher (DW) sent out email invitations where five individuals ultimately responded and agreed to review this paper. We present the comments from the 5 reviewers in Box 1. We conclude this paper with further discussions regarding when to conduct a patient-perspective analysis alongside some cautionary notes. Currencies were adjusted to 2020 using the annual Consumer Price Index and then converted to US dollars using purchasing power parities (11).

BOX 1 **Comments received from lived experience individuals.**

**Table 1:** 

Interventions	Comments
Interventions that cross the definitional boundary of drugs and non-drug technologies or replace prescription drugs	*“We too are often bewildered on how to engage these new “cross-program” interventions.”*
Interventions that significantly affect patient adherence to protocol by having unintended side effects	*“As a caregiver for a child with severe scoliosis, I endured a daily struggle to try to put him in a plastic back brace leaving me heartbroken as I just could no longer force him to wear it. I was also consumed with guilt because without the brace the alternative would be spinal surgery with all of the added risks involved.”* *“Adherence is ultimately in the sphere of influence controlled by the patient. It therefore runs completely counter-intuitive for patients not to be engaged in interventions that impact this critical nexus.”*
Interventions that represent revolutionary treatments or even cures for genetic disorders	*“Is the revolutionary intervention truly a cure or does it just hide the genetic disorder or just make it livable?”*
Interventions previously approved for coverage but now targeted for potential delisting or disinvestment	*“This is an area that I get most comments from other caregivers. They do not understand how their child, youth or adult can be deprived of a treatment that was working.”*

## 3. Types of health interventions that may warrant a patient-perspective analysis

### 3.1. Interventions that cross the definitional boundary of drugs and non-drug technologies or replace prescription drugs

The level of third-party coverage for drugs and non-drug technologies varies, most likely due to differing societal preferences for coverage and decision-making mechanisms. As one of the first countries to institutionalize HTA, Canada has operated a centralized evaluative process for recommending funding for brand-name drugs since 2003 through the Canadian Agency for Drugs and Technologies in Health’s Common Drug Review. Funding recommendations regarding non-drug technologies are made solely at the provincial level with notable variability in processes and decisions (12). This fragmentation creates issues for an emerging class of products that can neither be classified as a drug or non-drug technology. Patients who seek access to these products face financial obstacles as third-party coverage may have varying criteria for coverage; one of our reviewers shared this sentiment (Box 1). At the top of the scale, prescription drugs are generally covered within a single program budget with higher levels of coverage than within program budgets that cover medical devices (13). Thus, we believe funding decisions regarding these novel products might benefit from a separate patient-perspective analysis as it may highlight the potentially large increase in out-of-pocket costs. We present the examples of EndeavorRx, a game-based digital therapeutic devise approved in the US, and Abilify MyCite, a prescription drug with a digital ingestion tracking system, under this category.

The US Food and Drug Administration (FDA) has recently approved interventions that either replace prescription drugs or represent a hybrid of a drug and a medical device. An example of the first, EndeavorRx, was approved in 2020 as the first game-based digital therapeutic device prescribed for children with attention deficit hyperactivity disorder. This mobile device-based game involves navigating through a course with obstacles that is designed to improve attention function. EndeavorRx obtained FDA clearance through the *De Novo* Classification Pathway due to the lack of a predicate device on the market. To date, neither the FDA nor the manufacturer have released information on pricing and insurance coverage; hence, it is unknown what share of the cost families will face for this first-of-its-kind therapeutic device.

In 2017, Abilify MyCite was granted US market entry as the first prescription drug with a digital ingestion tracking system. Intended to treat adults with schizophrenia, bipolar disorder and depression, this combination product consists of Abilify (aripiprazole) tablets with an ingestible sensor that sends signal to a wearable patch communicating with a smartphone. Priced at 1,650 USD per month, Abilify MyCite is 80-times more costly than its drug-only counterpart and is covered in the US only for the most economically challenged (Medicaid beneficiaries). The cost-effectiveness of Abilify MyCite to the payer/society has yet to be established, but with its high out-of-pocket costs, it is unlikely to be affordable to many households without substantial third-party coverage.

### 3.2. Interventions that significantly affect patient adherence to protocol by having unintended side effects

A health intervention that is likely to impact adherence and priced substantially more than standard treatments warrants scrutiny within the entire HTA process. A common method to account for non-adherence in CEAs is through a small decrement in efficacy to produce a *de facto* measure of effectiveness; however, the decrement is commonly too small to result in any meaningful change in an incremental cost-effectiveness ratio (ICER) that would impact HTA recommendations (14). One of our reviewers, who are actively caring for a child wearing a medical device, expressed frustration in witnessing the side effects of the device and the lack of a more adherable and comfortable alternative (Box 1). Meanwhile, adherence-enhancing interventions usually demonstrate superior cost-effectiveness—if not cost-saving—from the societal or payer perspective (15). However, negative impacts on patient lives may remain essentially hidden within broader perspectives as there may be substantial benefits within these perspectives that could offset these negative impacts to patients; therefore, patient-perspective evaluations are needed to reveal potentially important patient-level effects; one of our reviewers concurred (Box 1).

One example of such an intervention—FreeStyle Libre—is a wearable flash glucose monitoring system for people with diabetes as an alternative to finger-prick tests. The system comprises a disposable subdural sensor and a device that receives and stores data. In 2020, the Ontario Health Technology Advisory Committee (OHTAC) and the HTA Advisory Board of Quebec have both recommended funding FreeStyle Libre, despite analyses showing that it may not be cost-effective to payers compared to finger-prick tests (16). A recommendation to fund the intervention was partially driven by feedback from patients and caregivers who reported greater ease of use permitting them greater control over their lives. This helped to overcome uncertainty over its cost-effectiveness to improve blood glucose stability.

While Abilify MyCite has already been mentioned, there is a general class of drug-device hybrid products called digital pills emerging that combine prescription drugs with an ingestible sensor, a wearable patch and a mobile application (17). By continuously tracking patients’ medication-taking behaviours, digital pills aim to improve patient adherence, help forge self-care routines and enhance patient-physician relationships. However, patients report fatigue and disruption of daily routines by going through extensive training to correctly operate the wearable patch and to successfully pair it with a smartphone. This imposition on patient lives may be greater than the burdens of manually remembering to take medications thereby defeating the marginal benefit associated with using digital pills (18). Another issue is related to privacy, that is, who would be authorized to access patient data. At the extreme, these concerns may be disruptive leading to a refusal to take the medication. Furthermore, there is the danger of device-associated emergent adverse events that have implications for both patient well-being and adherence to protocol (17). These uncertainties raise questions regarding willingness-to-pay out-of-pocket for a product carrying such risks for improvements in adherence. A formal patient-perspective analysis may be able to provide answers by soliciting inputs that highlight these uncertainties.

Nirmatrelvir with ritonavir, sold under the brand name Paxlovid, is another example of interventions that fall under this category. In December 2021, the US FDA approved the use of Paxlovid for treating mild-to-moderate COVID-19 under an emergency authorization. Soon after its approval, reports of side effects started to emerge, most notably regarding the bitter metallic taste Paxlovid sometimes left in mouth (“Paxlovid mouth”). Recent studies of “Paxlovid rebound,” which refers to an asymptomatic or symptomatic resurgence of COVID-19 after finishing the full 5-day course of Paxlovid, suggest this may be due to patients skipping a dose to avoid the unwanted aftertaste of Paxlovid (19, 20). This conjecture requires more research to confirm.

### 3.3. Interventions that represent revolutionary treatments or even cures for genetic disorders

Recent breakthroughs have enabled new treatments for genetic disorders that previously were considered untreatable. In clinical trials, these treatments demonstrate high incremental effectiveness or even a cure. While revolutionary, they may place added burdens on patients’ lives or produce inadequately measured risks. Indeed, one of our reviewers raised concern on the real-world outcome of these treatments, and specifically, if a cure was truly attainable (Box 1). In this case, we suggest that a separate patient-perspective analysis that elicits inputs from patients and their families might provide important insights to support informed decision-making. We talk about treatments for spinal muscular atrophy as an example.

In 2017, Spinraza (nusinersen) was approved by the US FDA and the European Medicines Agency for the treatment of spinal muscular atrophy, a group of rare, genetic neuromuscular disorders that leads to severe muscle weakness and progressive loss of motor function. In 2020, The Japanese Ministry of Health, Labour and Welfare approved Zolgensma (Onasemnogene abeparvovec), a single-dose intravenous gene replacement therapy that replaces Spinraza’s four loading doses. Compared to Spinraza, Zolgensma is less costly (2 million USD vs. 2.2–10.6 million USD for Spinraza over a lifetime) and is potentially superior to Spinraza by reducing treatment complexity (21). However, patient representatives have voiced concerns on the durability of the long-term benefits of Zolgensma and on the uncertainty of treatment pathways if gene expression diminishes over time (21). Furthermore, to help better understand the particulars of treatment protocols, patients may value genetic counseling, but the availability of such counseling service is often limited in real-world clinical settings—a usually unmeasured shortcoming. None of these effects are captured within current analyses.

### 3.4. Interventions with an expected ICER in the southwest quadrant of the cost-effectiveness plane from a payer or societal perspective

Funding decisions are often difficult for interventions that are less costly but slightly less effective from payer/societal perspectives than currently utilized therapies. Debates have been on the extent to which the loss of a quality-adjusted life year (QALY) differs from the value of acquiring a QALY. Review studies found Willingness-To-Accept/Willingness-To-Pay ratios among health interventions to range from 1.9 to 6.4 (22). These observations point to a kink in consumer threshold values where patients are generally more reluctant to lose than they are willing to gain. Currently, there is a lack of consensus on how to address this potential asymmetry (23). Hence, we argue that when confronted with this situation, a patient-perspective analysis in which patient-level costs are weighted more heavily might help guide funding decisions.

In 2002, the National Institute for Health and Care Excellence (NICE) recommended vinorelbine as one option for second-line treatment for advanced breast cancer (24). The only UK-based CEA at that time concluded vinorelbine to be slightly inferior to taxanes in terms of quality of life improvement, while being markedly cheaper. The same study found the ICER of vinorelbine to be 37,277 USD/QALY (14,500 pounds/QALY in 1998 values) and 5,116 USD/QALY (1,990 pounds/QALY in 1998 values)—in the southwest quadrant—when compared to docetaxel and paclitaxel, respectively. Taking into account the patient perspective, NICE recommended to keep funding vinorelbine due to its safety and tolerability among patients distinguishing vinorelbine as a more patient-friendly option than taxanes.

An internet-mediated cognitive behavioral therapy for treating mild to moderate depression is a more recent example (25). Trial results suggest that this Swedish-recommended intervention produced 0.05 fewer QALYs over 12 months while saving 326 USD (2,664 SEK in 2013 values) for the society compared to usual care (primary care physician visits, nurse visits, antidepressants, face-to-face psychotherapy and/or sick leave). The study concluded that this therapy is at least as cost-effective as usual care and that the choice of treatments ultimately relied on patient preferences regarding ease-of-use, availability and willingness to wait for services.

### 3.5. Interventions previously approved for coverage but now targeted for potential delisting or disinvestment

Third-party payers sometimes must make decisions to delist a previously reimbursed intervention. This may have profound and sometimes unintended consequences to patients. According to one of our reviewers, caregivers are usually baffled by the delisting of a health intervention that has been working for the patient (Box 1). It is imperative that, prior to delisting, the perspective of patients’ needs to be considered to avoid harm to subsets of patients. Furthermore, a grace period during which patients can be phased out of these interventions and transition into a new protocol should be clearly defined and structured. We argue that a patient-perspective analysis may help clarify the relative costs and benefits that will befall patients.

In 2017, the OHTAC recommended discontinuing public funding for external cardiac loop recorders (ELR) for diagnosing cardiac arrhythmia if the device relied solely on patient-initiated recordings. In consideration of the recommendation, the Ontario Ministry of Health now funds ELR only if it is operated by a cardiologist and funding also continues for long-term continuous ambulatory electrocardiogram monitors (ECGm), a more advanced alternative (26). These decisions were made on the basis that, despite a small annual increase in provincial expenditures due to increased diffusion of ECGm, these expenses were justified given incremental improvements in patient experiences. However, cardiologists voiced concerns regarding the disinvestment decision as ELR and ECGm had been traditionally used to diagnose different patient populations—one that is able to self-monitor and the other monitored by a cardiologist, respectively. Hence, a phasing out of reimbursement for ELR reduces patient access to a proper diagnosis as a limited supply of cardiologists must now supervise ELR testing (27). This issue cannot be overlooked because adoption and disinvestment policies need solid implementation plans, including potential grace periods, to manage unintended consequences in both cases.

These unintended effects of disinvestment on patients and their caregivers should be better anticipated. For example, expectorants and mucolytics, that make coughing up mucus easier and less irritating, were dropped in France from the publicly funded formulary in 2006 as a physician’s prescription was no longer needed. The resulting over-the-counter market for these products saw price increases of up to 200%, the full cost of which was borne by patients leading to affordability issues for those with lower incomes (28). A second example involved the delisting of phlebotonics for chronic venous diseases in Italy that was followed by an increase in hospitalizations for venous insufficiency (29). Perhaps, better consultations with experts could assist implementation to either soften or prevent the adverse effects noted.

## 4. Discussion

Existing health economic evaluation studies using the patient’s perspective rarely provide a clear rationale on the conduct of a focused patient-centred examination (4). As such, the preceding has been a listing of situations in which patient-level assessment of both affordability and level of imposition on patient lives is warranted alongside a societal/payer perspective analysis to partially address equity concerns. In terms of affordability, many jurisdictions provide full coverage for those under the poverty line. For others, a limit is set on out-of-pocket costs between 1% to 7.5% of income in order to qualify for either tax deductions or credits, and/or government programs that provide catastrophic coverage (30, 31). Thus, affordability varies by level of income under the current patchwork system (32). A marginal increase of at least 1% of income over standard praxis might be worthy of notice for any subset of patients based on the base threshold set for tax deductibility of medical expenses in Germany, the lowest amongst western countries (31). No similar threshold for imposition on patient lives neither exists nor is recommended other than to suggest assessing the impacts individually for each intervention.

Though perhaps not exhaustive, the list of situations provided represent a first attempt to elucidate a shortcoming in current economic evaluation guidelines. We have shown that there is the potential for substantial incongruencies in findings between narrower economic analyses from the perspective of patients than broader analyses. These differences in findings may suggest that net effects in these broader perspectives tend to hide details that may be important from the point-of-view of decision-making bodies. By weighing all costs equally, these broader analyses may be underestimating some cost components that these bodies may wish to weigh more heavily prior to concluding about whether and how to move forward. We believe HTA agencies need to actively incorporate patient inputs throughout the entire HTA process, but until we reach expert consensus on how to quantitatively account for these data in cost-effectiveness analyses, more feasible options include a qualitative literature review about patient preferences and/or direct engagement activities (such as a focus group discussion) with these individuals (33). Caution must be taken, however, to not double-count time/adherence costs that may already be captured within QALY decrements and therefore should not be added to financial costs. At this juncture, more work is needed to explicate the various situations in which a patient-perspective economic evaluation is not only warranted but potentially recommended.

## Data availability statement

The original contributions presented in the study are included in the article/supplementary material, further inquiries can be directed to the corresponding author.

## Author contributions

RF, VN, ML, DW, EY, and EN: conceptualization, investigation, and writing—review and editing. RF, VN, and EN: methodology. RF: writing—original draft preparation. EN: supervision. All authors contributed to the article and approved the submitted version.

## Conflict of interest

VN was employed by Roche Diagnostics.

The remaining authors declare that the research was conducted in the absence of any commercial or financial relationships that could be construed as a potential conflict of interest.

## Publisher’s note

All claims expressed in this article are solely those of the authors and do not necessarily represent those of their affiliated organizations, or those of the publisher, the editors and the reviewers. Any product that may be evaluated in this article, or claim that may be made by its manufacturer, is not guaranteed or endorsed by the publisher.
